# Serogroup W135 Meningococcal Disease, The Gambia, 2012

**DOI:** 10.3201/eid1909.130077

**Published:** 2013-09

**Authors:** M. Jahangir Hossain, Anna Roca, Grant A. Mackenzie, Momodou Jasseh, Mohammad Ilias Hossain, Shah Muhammad, Manjang Ahmed, Osuorah Donatus Chidiebere, Ndiaye Malick, S.M. Bilquees, Usman N. Ikumapayi, Baba Jeng, Baba Njie, Mamady Cham, Beate Kampmann, Tumani Corrah, Stephen Howie, Umberto D’Alessandro

**Affiliations:** Medical Research Council Unit, Banjul, The Gambia (M.J. Hossain, A. Roca, G.A. Mackenzie, M. Jasseh, M.I. Hossain, S. Muhammad, M. Ahmed, O.D. Chidiebere, N. Malick, S.M. Bilquees, U.N. Ikumapayi, B. Kampmann, T. Corrah, S. Howie, U. D’Alessandro);; Ministry of Health, Banjul (B. Jeng, B. Njie, M. Cham);; Institute of Tropical Medicine, Antwerp, Belgium (U. D’Alessandro)

**Keywords:** Neisseria meningitidis, serogroup W135, meningitis, the Gambia, epidemic, outbreak, bacteria

## Abstract

In 2012, an outbreak of *Neisseria meningitidis* serogroup W135 occurred in The Gambia. The attack rate was highest among young children. The associated risk factors were male sex, contact with meningitis patients, and difficult breathing. Enhanced surveillance facilitates early epidemic detection, and multiserogroup conjugate vaccine could reduce meningococcal epidemics in The Gambia.

Meningococcal disease is endemic to the African “meningitis belt”; outbreaks occur regularly (*1*,*2*). *Neisseria meningitidis* serogroup A causes most (80%) cases. However, during 2002–2003, serogroup W135 caused a major epidemic in Burkina Faso (attack rate [AR] 251 cases/100,000 population) (*3*). Thereafter, the incidence of serogroup W135 was low, with isolated cases and a small-scale outbreak in the meningitis belt (*4*,*5*). In 2010, serogroup A conjugate vaccine was introduced into the African meningitis belt and substantially reduced the incidence of meningitis (*6*).

In The Gambia, only 6 serogroup W135 cases were identified during 1990–1995; the most recent case had been reported in 1995 (*7*). In 2012, a large epidemic of serogroup W135 occurred throughout the meningitis belt, including The Gambia (*1*). Most risk factors identified in the meningitis belt concern serogroup A (*8*,*9*), and risk factors for serogroup W135 are little studied. Therefore, we report the investigation of this epidemic and the related risk factors.

## The Study

The Gambian Ministry of Health and the Medical Research Council Unit, The Gambia, investigated a serogroup W135 epidemic that occurred during February–June 2012 in the Central River Region (CRR) and Upper River Region (URR). Since 2008, surveillance of invasive bacterial diseases has been ongoing in Bansang Hospital in CRR and Basse Health Centre in URR (*10*). The peripheral health centers refer severely ill patients to these health facilities. Three approaches were used to recruit persons with suspected cases of serogroup W135: enhanced prospective surveillance in Bansang Hospital and Basse Health Centre, retrospective case identification from hospital records, and visits to households with confirmed case-patients serogroup W135 to identify other suspected cases. A suspected case was defined as a history of acute onset of fever and any of the following: altered consciousness, inability to eat, neck stiffness, seizures, petechial rash, or bulging anterior fontanel in a child <2 years of age. Cerebrospinal fluid (CSF) and/or blood samples were collected from hospitalized persons with suspected serogroup W135. A confirmed case was a suspected case in which serogroup W135 was identified by culture and/or an antigen-specific test. The alert threshold was defined as >5 meningitis cases per 100,000 persons per week; the epidemic threshold was >10 cases (*11*).

The investigation team administered 1 dose of ciprofloxacin to each close contact of confirmed case-patients and provided health information to raise awareness. At the end of the epidemic, The Gambian government deployed the tetravalent meningococcal polysaccharide vaccine.

CSF and blood samples were cultured for bacteria in BACTEC Medium (Becton Dickinson, Franklin Lakes, NJ, USA) and tested for serogrouping by latex agglutination by using BACTEC and Ramel (Thermo Fisher Scientific, Waltham, MA, USA) test kits. Antimicrobial drug susceptibility was tested.

We conducted a matched case–control (ratio 1:1) study to identify risk factors. Healthy controls were matched by age and village with confirmed case-patients, including those who died. Demographic, socioeconomic, and exposure (within 14 days before illness onset) data were collected by using a structured questionnaire. Risk factors were analyzed by conducting bivariate matched and multivariate conditional logistic regression analyses. The Joint Gambia Government/Medical Research Council Ethics Committee approved the study. All study participants or legal guardians provided written informed consent.

During February 1–June 25, 2012, a total of 469 suspected cases were identified, and 114 were confirmed to be serogroup W135. Thirty-one were co-primary or secondary cases in confirmed case-patients’ households. Most (67%) suspected case-patients were <5 years of age, and 56% of cases occurred in male patients. The overall case-fatality rate was 8%.

**Figure 1:**
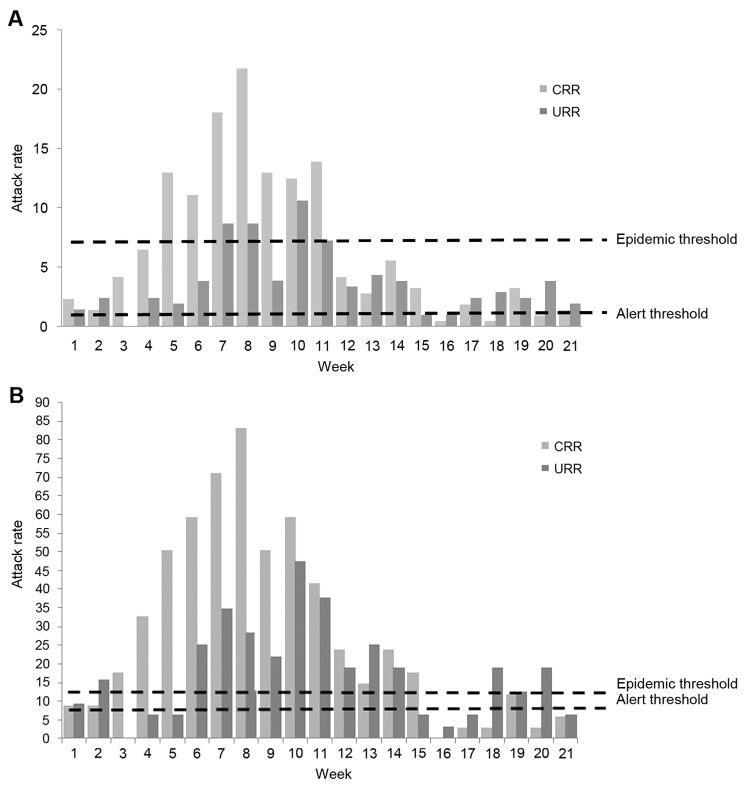
Outbreak of *Neisseria meningitidis* serogroup W135, Central River and Upper River Regions, The Gambia, February 1–June 25, 2012. A) Attack rate per 100,000 persons per week. B) Attack rate per 100,000 children <5 years of age per week. Light gray bars, Central River Region; dark gray bars, Upper River Region. Alert threshold corresponds to an attack rate of 5; epidemic threshold, to an attack rate of 10.

**Figure 2:**
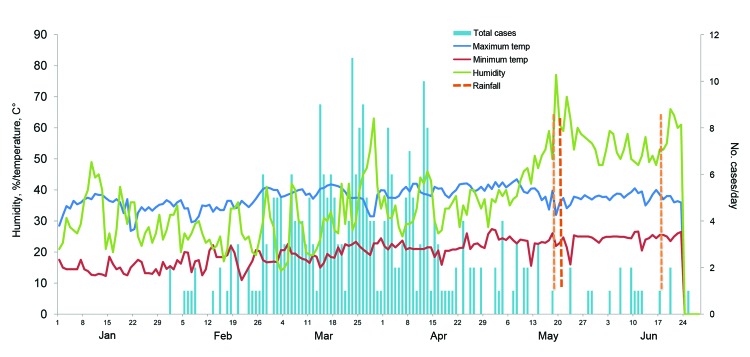
Number of epidemic cases of *Neisseria meningitidis* serogroup W135 in relation to time, temperature, humidity, and rainfall, Central River Region, The Gambia, February 1–June 25, 2012. Blue line, maximum temperature; red line, minimum temperature; green line, humidity; blue bars, total number of cases.

The overall AR was 111 cases per 100,000 persons but was much higher among younger children (Table 1). The epidemic threshold was exceeded in the last week of February and continued until April for persons of all ages and until June for children <5 years of age (Figure 1, panels A, B). Among children <5 years of age, the peak AR attained 83 and 47 cases per 100,000 persons per week in CRR and URR, respectively (Figure 1, panel B). The epidemic peaked during the high temperature/driest months (March–May) and ended abruptly after the first rainfalls in June (Figure 2).

**Table 1:** Confirmed and suspected cases of *Neisseria meningitidis* serogroup W135, CRR and URR, The Gambia, February 1–June 25, 2012*

Health region/case-patient age group, y	Cases, no. (%)	Deaths, no. (%)	2011 population†	Cases/100,000 population
CRR				
<1	62 (20)	8 (13)	4,216	1,470
1–4	138 (45)	4 (3)	29,470	468
5–14	70 (23)	2 (3)	66,545	105
>15	37 (12)	4 (11)	115,995	32
Total	307 (100)	18 (6)	216,227	142
URR				
<1	47 (29)	10 (21)	4,086	1,150
1–4	70 (43)	5 (7)	27,564	254
5–14	36 (22)	2 (6)	64,014	56
>15	9 (6)	1 (11)	111,663	8
Total	162 (100)	18 (11)	207,327	78
CRR and URR				
<1	109 (23)	18	8,302	1,312
1–4	208 (44)	9	57,034	364
5–14	106 (23)	4 (4)	130,560	81
>15	46 (10)	5 (11)	227,658	20
Total	469 (100)	36 (8)	423,554	111

*CRR, Central River Region; URR, Upper River Region. †Estimated on the basis of 2003 census.

The most common signs and symptoms among the 113 confirmed serogroup W135 case-patients were weakness (96%), irritability (88%), neck stiffness (81%), and inability to eat (80%). Bulging fontanelle (74%), altered mental status (73%), and seizures (65%) occurred in a slightly lower proportion of case-patients.

Blood and/or CSF samples were collected from 301 (69%) of 438 hospitalized suspected case-patients, of which almost half (138) were positive for bacterial pathogens. Serogroup W135 was the major pathogen (114 [83%] of 138); followed by *Streptococcus pneumoniae* (13%) and *Staphylococcus aureus* (2%). Common antibacterial drugs used for meningitis were tested on 92 (81%) serogroup W135 isolates, which were susceptible to most of them (ampicillin, 100%; chloramphenicol, 99%; ciprofloxacin, 99%; penicillin, 95%; and tetracycline, 92%) but not to erythromycin (59% susceptible) and trimethoprim/sulfamethoxazole (100% resistant).

We enrolled 106 confirmed case–control pairs. Risk factors identified in the univariate analysis were male sex, students, >4 children 1–5 years of age in the household, contact with a meningitis case-patient, preceding history of respiratory illness (nasal discharge, difficult breathing), and itchy eyes (Table 2, Appendix). In the multivariate analysis, male sex (odds ratio [OR] 1.9; 95% CI 1.0–3.7), contact with meningitis case-patients (OR 4.8; 95% CI 1.3–17.8), difficult breathing (OR 6.8; 95% CI 1.4–33.4), and itchy eyes (OR 4.4; 95% CI 1.3–14.4) remained significantly associated with cases.

**Table 2:** Matched univariate analysis for risk factors for *Neisseria meningitidis* serogroup W135 during an outbreak, Central River Region and Upper River Region, The Gambia, February 1–June 25, 2012

Characteristic	Cases no. (%), n = 106	Controls, no. (%), n = 106	Odds ratio (95% CI)	p value
Demographic				
Mean age (±SD), y	4 (3.9)	3.9 (3.7)		0.876*
Male sex	66 (62)	53 (50)	1.7 (0.96–3.1)	0.067
Ethnic group				
Mandinka	33 (31)	31 (29)	Ref	
Sarahule	13 (12)	13 (12)	0.80 (0.5–13.7)	0.881
Fula	44 (42)	39 (37)	0.64 (0.2–1.9)	0.421
Wollof	17 (16)	20 (19)	1.6 (0.3–7.7)	0.567
Other	1 (01)	1 (1)	1.0 (0.1–21.5)	0.993
Socioeconomic				
Occupation: student	22 (21)	14 (13)	5.0 (1.1–22.8)	0.038
Mean age of student (±SD), y	9.8 (4.7)	9.6 (3.9)		0.897*
Primary caretaker: mother	92 (87)	95 (90)	0.8 (0.3–1.8)	0.523
Occupation: farming	66 (62	68 (64)	0.9 (0.5–1.7)	0.73
Formal schooling: none	90 (85)	83 (78)	1.5 (0.8–3.1)	0.227
Housing				
Floor earth or sand	42 (40)	42 (40)	1.0 (0.5–1.9)	0.769
Roof with thatched	36 (34)	46 (43)	0.57 (0.3–1.1)	0.158
Wall with mud	68 (64)	74 (70)	0.63 (0.3–1.4)	0.381
Household item				
Television	22 (21)	20 (19)	1.2 (0.5–2.5)	0.695
Bicycle	82 (77)	78 (74)	1.3 (0.6–2.6)	0.481
Tube well for drinking water	34 (32)	31 (29)	1.5 (0.5–4.2)	0.442
Traditional pit latrine	98 (92)	98 (92)	1.0 (0.3–3.1)	1.0
Travel within 14 d before illness onset				
Hospital or health center	19 (18)	23 (22)	0.76 (0.37–1.6)	0.467
Outside the village	24 (22)	23 (22)	1.06 (0.55–2.1)	0.866
Festival	21 (20)	18 (17)	1.2 (0.60–2.4)	0.602
Household crowding				
>1 household in participant’s compound	39 (37)	40 (37)	1.05 (0.57–1.9)	0.876
Household member				
Median (interquartile range)	20 (5–79)	18 (4–51)		0.412
>20 persons in household	50 (47)	40 (38)	1.7 (0.88–3.3)	0.109
Household member by age				
>1 children >1 y	72 (68)	66 (62)	1.3 (0.72–2.5)	0.356
>4 children 1–5 y	59 (56)	48 (45)	2.1 (1.1–4.1)	0.037
<5 persons in sleeping room	25 (24)	25 (24)	1.0 (0.46–2.2)	1.00
>1 window in sleeping room	74 (70)	64 (60)	0.44 (0.19–1.0)	0.056
>3 persons sharing same room	68 (64)	64 (60)	1.2 (0.67–2.0)	0.572
>1 person sharing same bed	99 (93)	103 (97)	0.33 (0.07–1.6)	0.178
Social crowding in 14 d before illness onset				
Traveled in public transport in a week >1 d	8 (8)	15 (14)	0.54 (0.21–1.4)	0.187
Attended gathering larger than the no. persons living in participant’s compound	37 (32)	32 (30)	1.1 (0.59–2.1)	0.746
Attended school	20 (19)	14 (13)	2.5 (0.78–8.0)	0.103
Concurrent or recent respiratory or other illness within 14 d before illness onset				
Cough	43 (41)	41 (39)	1.1 (0.59–2.1)	0.746
Nasal discharge	50 (47)	30 (28)	3.0 (1.5–6.1)	0.003
Sore throat	11 (11)	6 (6)	2.0 (0.68–5.9)	0.206
Difficult breathing	17 (16)	5 (5)	7.0 (1.6–30.8)	0.010
Itchy eyes	18 (17)	4 (4)	4.5 (1.5–13.3)	0.007
Itchy nose	10 (10)	3 (3)	3.3 (0.91–12.1)	0.067
Itchy throat	4 (4)	3 (3)	1.3 (0.03–5.9)	0.706
Ear infection	9 (9)	6 (6)	1.6 (0.52–4.9)	0.410
Diarrhea	36 (34)	27 (26)	1.8 (0.86–3.6)	0.122
Symptoms of respiratory infection (cough, nasal discharge or difficult breathing)	61 (58)	53 (50)	1.5 (0.80–2.8)	0.209
Contact with patients who have symptoms of meningoencephalitis within 14 d before illness onset				
Knew any person with fever with convulsion, altered mental status or unconsciousness	22 (21)	18 (17)	1.4 (0.62–3.2)	0.416
Contact (within 1 m) with any person with symptoms of meningoencephalitis	15 (15)	6 (6)	4.0 (1.1–14.1)	0.032
Site of contact				
No contact	91 (86)	100 (94)	1	
Contact outside compound	8 (8)	4 (4)	2.9 (0.70–12.4)	0.142
Contact in household and compound	7 (7)	1 (2)	7.4 (0.84–65.0)	0.072
Exposure to smoke within 14 d before illness onset				
Any person smoked cigarette or other tobacco products in household	58 (55)	57 (54)	1.04 (0.58–1.9)	0.882
Kitchen inside living room or veranda	5 (5)	2 (2)	4.0 (0.45–35.8)	0.215
Exposed to cooking smoke during cooking	86 (81)	78 (74)	1.7 (0.82–3.6)	0.149
Carried children (<2 y) on the back whiule cooking, n = 74	24 (65)	20 (54)	1.6 (0.60–4.6)	0.323
Used mosquito repellent or fires to keep body warm	10 (9.4)	5 (4.7)	2.7 (0.71–10.1)	0.147
Child currently breast-feeding	31 (29)	37 (35)	0.25 (0.05–1.2)	0.080

*p value by Wilcoxon rank-sum (Mann-Whitney) test.

## Conclusions

Before the current cases, the most recent sporadic cases in The Gambia were reported in the early 1990s. These cases were part of a larger epidemic in the meningitis belt with a comparable predominance of serogroup W135 followed by *S. pneumoniae* (*1*). After introduction of MenAfriVac (serogroup A conjugate vaccine), incidence and epidemics caused by serogroup A decreased substantially in the meningitis belt (*6*,*12*). The reemergence of epidemic serogroup W135 in this region requires a strategy for surveillance, epidemic detection and control, and revised vaccination policy.

Serogroup A outbreaks usually affect children >5 years of age and young adults (*5*,*13*,*14*). However, two thirds of the serogroup W135 cases occurred in children <5 years of age, for whom the AR was 5-fold higher than it was for older age groups, similar to the characteristics of the serogroups W135 outbreaks in Burkina Faso (2002–2003) and Niger (2010) (*3*,*5*). Therefore, the current operational definition of alert and epidemic thresholds, drawn mostly from serogroup A data, should be revised because merging the AR for all age groups may delay, or result in nondetection of, a serogroup W135 epidemic in younger age groups.

Signs and symptoms of concurrent respiratory illness were more prevalent among case-patients than controls;, itchy eyes and difficult breathing were associated with disease. The temporal sequence of these signs relative to the occurrence of meningococcal disease was not determined, and whether these factors facilitated the invasion of serogroup W135 carried in the nasopharynx or whether these symptoms were part of the initial serogroup W135 infection before onset of severe disease is unclear. Contact with confirmed serogroup W135 case-patients was a strong risk factor. These results are consistent with information available for the other serogroups and with the route of serogroup W135 transmission through droplet infection (*15*).

Our findings suggest that isolation of case-patients and prophylactic treatment of contacts may reduce transmission of meningococcal disease during epidemics. Enhanced surveillance for meningitis is recommended for early detection of epidemics. The occurrence of this large serogroup W135 outbreak suggests that multiserogroup conjugate vaccine should be deployed for control and prevention.
